# Use of Bioelectrical Impedance Analysis to Measure the Impact of Parasitic Infection on Goat Sperm Quality

**DOI:** 10.3390/ani15243624

**Published:** 2025-12-17

**Authors:** Abdallah M. Shahat, Ranadheer Narlagiri, Aftab Siddique, Sai Chandan Chelkapally, Ramya Sri Kolikapongu, Sharath Chandra Namani, Arshad Shaik, Phaneendra Batchu, Priyanka Gurrapu, Tharun Tej Erukulla, Ayesha Neha, Thomas H. Terrill, Adel R. Moawad

**Affiliations:** 1 Animal Science Program, College of Agriculture, Family Sciences, and Technology, Fort Valley State University, Fort Valley, GA 31030, USA; 2 Department of Theriogenology, Faculty of Veterinary Medicine, Cairo University, Giza 12211, Egypt

**Keywords:** goats, parasitic infection, testicle, spermatozoa, bioelectrical impedance

## Abstract

Parasitic infection is a major cause of infertility in small ruminants. This study aimed to assess the association between bioelectrical impedance analysis (BIA) measurements, testicular morphometrics, and sperm quality in parasitized goats. Thirty-eight intact mature Spanish bucks were allowed to graze on a naturally parasitically infected pasture for 3 months. Nineteen bucks were dewormed regularly (healthy group), while the other 19 bucks did not receive any anthelmintics (parasitized group). Fecal and blood samples were collected weekly to assess fecal egg count (FEC) and packed cell volume (PCV), respectively. Based on the size and morphology of the parasite eggs, they were presumptively identified as *Haemonchus contortus.* At the end of the grazing period, bucks were slaughtered, and testicles and epididymis were collected for analysis. In addition, BIA was applied to each testicle to measure series resistance (Rs) and reactance (Xc). Epididymal spermatozoa were retrieved and evaluated for motility, viability, morphology, and membrane and acrosome integrities. Data was analyzed using the Mann–Whitney U and Pearson Correlation Coefficient tests. The results showed that Rs (169.41 ± 1.76 Ω vs. 235.21 ± 20.21 Ω), Xc (37.55 ± 0.48 Ω vs. 52.08 ± 4.68 Ω), testicular and epididymis weights and lengths, sperm motility, viability, and membrane and acrosome integrities were lower (*p* < 0.0001) in parasitized than in healthy goats. Strong correlations (*p* < 0.001) were observed between Rs, sperm viability (r = 0.20), membrane integrity (r = 0.15), and acrosome integrity (r = 0.14), as well as between Xc and the same sperm parameters (r = 0.21, 0.18, and 0.16, respectively). In conclusion, our findings demonstrate that parasitic infection is associated with testicular health and subsequent epididymal sperm quality of goats. BIA can be utilized as an efficient tool to predict the impact of parasitic infection on testicular function in goats.

## Simple Summary

The association between bioelectrical impedance analysis (BIA) measurements and sperm quality parameters in parasitized goats was assessed in this study. Thirty-eight Spanish male goats were split evenly into two groups: namely, parasitized (average fecal egg count (FEC) = 517.4 ± 0.16 and average packed cell volume (PCV) = 17.65 ± 4.13) and healthy (average FEC = 47.2 ± 0.26 and average PCV = 25.92 ± 4.47) ones. Healthy goats were free of parasites due to routine deworming; meanwhile, parasitized goats contracted the infection by grazing untreated pasture for 3 months. Following slaughter, testicles and epididymis were collected, and their weights and lengths were recorded. BIA was then applied to the testicles to record the series resistance (Rs) and reactance (Xc), as well as other BIA measurements. Epididymal sperm were retrieved to assess motility, viability, morphology, and membrane and acrosome integrities. We found that BIA measurements, testicular morphometrics, and sperm quality parameters were lower in parasitized goats than in healthy ones. Strong correlation was also seen between Rs and Xc values and sperm parameters. These findings suggest that parasitic infection of goats has adverse effects on testicular health and sperm quality, which may negatively impact male fertility. The results also demonstrate that BIA could be a promising noninvasive tool for predicting these effects.

## 1. Introduction

Goat production is an important livestock industry throughout the world [1]. Since the early 1990s, the United States has developed a strong interest in meat goat production, and since then, meat goat production has spread all over the country [2]. However, this productivity has been severely undermined by gastrointestinal (GI) parasites, which are a leading cause of diseases and economic losses in small ruminants [3,4,5]. A variety of endo- or ecto-parasites can affect these animals, and their adverse effects on health, production, and welfare have been documented [6]. Within this frame, trichostrongylid gastrointestinal infections currently are among the major challenges in goat health management due to prevalent anthelmintic resistance in many parts of the world [6], which increases the potential harmful effects on the health and welfare of the animals.

Testes size is a critical component in male reproductive success [7], given that it has a significant correlation to daily sperm production and fertility in males [8]. Parasitic infestations can directly or indirectly impair testis function, causing infertility [9,10]. In sheep, it has been shown that infection with *Haemonchus contortus*, a hematophagous gastrointestinal parasite, significantly reduced sperm cell concentration and sperm viability compared to those seen in healthy rams [9]. Furthermore, other studies have shown a decrease in plasma testosterone concentrations in rams infected with *Haemonchus contortus* compared to controls [9,11]. These findings highlight a correlation between parasitic infestation and reproductive traits in males [9].

The development of rapid, noninvasive tools to assess male reproductive health is, therefore, crucial for effective livestock management. Bioelectric impedance analysis (BIA) is a validated alternative method that uses surface electrodes to apply electrical energy and measure tissue responses, including series resistance (Rs) and reactance (Xc) [10]. BIA relies on Ohm’s law, which states that the voltage across a conductor is proportional to its resistance to current flow [12]. When an electrical current is applied to biological tissue, its components promote the charge transfer. Mobile ions in water are the primary charge carriers, while proteins and lipidic cell membranes carry positive and negative charges [12]. Measuring electric characteristics provides insight into the properties and composition of tissues [10].

Numerous studies have demonstrated the prognostic value of raw bioelectrical parameters in patients with Human Immunodeficiency Virus (HIV) infection, cancer, conditions necessitating hemodialysis, malnutrition, and anorexia nervosa, indicating their potential applications for clinicians [13,14]. Cross-sectional studies have revealed moderately positive correlations between BIA phase angle (PA) and fat-free mass, as well as significant differences in Rs, Xc, and PA between well-trained bodybuilders and healthy controls, suggesting that variables like PA also seem to distinguish between people with high or low levels of muscle mass [15,16]. Although many investigations have reported the cross-sectional associations of BIA parameters with aspects of health, disease, and physical performance, limited information is available on utilizing BIA for predicting sperm quality in bucks subjected to parasitic diseases. In humans, a recent study investigated the potential association between the PA of BIA and sperm quality in infertile males [17]. The findings of this study suggested that patients with lower PA had impaired sperm quality, particularly lower sperm concentration and total sperm count [17].

The association between BIA measurements, sperm quality, and male fertility in livestock remains entirely unexplored. Herein, we hypothesized that changes induced in testicular tissue by parasitic infection would alter its bioelectrical properties and that these changes would be correlated with standard measures of testicular morphometrics and sperm traits. Therefore, the aim of the current study was to assess the association between BIA, testicular morphometrics, and sperm quality in parasitized goats.

## 2. Materials and Methods

### 2.1. Animals

All animal protocols were approved by the Fort Valley State University (FVSU, Fort Valley, GA, USA) Agricultural and Laboratory Animal Care and Use Committee (ALACUC approval number WI-R-02-23).

**Figure 1:**
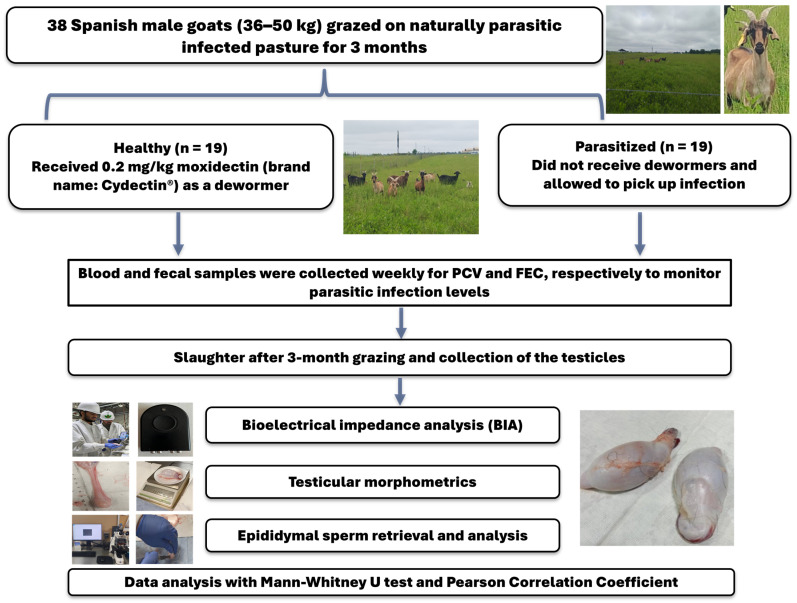
Schematic diagram showing the experimental approach. PCV: packed cell volume; FEC: fecal egg count.

Thirty-eight intact Improved Spanish male goats (24 months old; 36–50 kg) purchased from Whitworth Ranch Junction (Kimble County, TX, USA) grazed the same grass pasture at the FVSU Agriculture Technology Center farm from September to December 2023. From these bucks, 19 were frequently received 0.2 mg/kg of moxidectin (brand name: Cydectin^®^, Elanco, Greenfield, IN, USA) as a dewormer, and they were categorized as the healthy (H) group. The other 19 males did not receive any dewormers and were allowed to pick up a natural parasitic infection, and they were identified as the parasitized (P) group. Fecal and blood samples were collected weekly from each male to assess fecal egg count (FEC) and packed cell volume (PCV), respectively, to screen the levels of parasitic infestation [18]. Fecal samples were processed and examined under a compound microscope (Fisher Scientific, Atlanta, GA, USA) with a 100× magnification lens with a camera mounted on the eyepiece (1600× digital zoom). The size of the eggs was compared to a calibrated reference image with a 50 μm scale bar. Based on morphology, regional prevalence, and clinical signs (anemia), the infections were presumptively attributed to *Haemonchus contortus* eggs [19]. The average FEC and PCV values in the healthy group were 47.2 ± 0.26 and 25.92 ± 4.47, respectively, while in the parasitized group, they were 517.4 ± 0.16 and 17.65 ± 4.13, respectively. A schematic diagram showing the experimental approach is presented in Figure 1.

### 2.2. Collection of Testicles and Application of BIA

**Figure 2:**
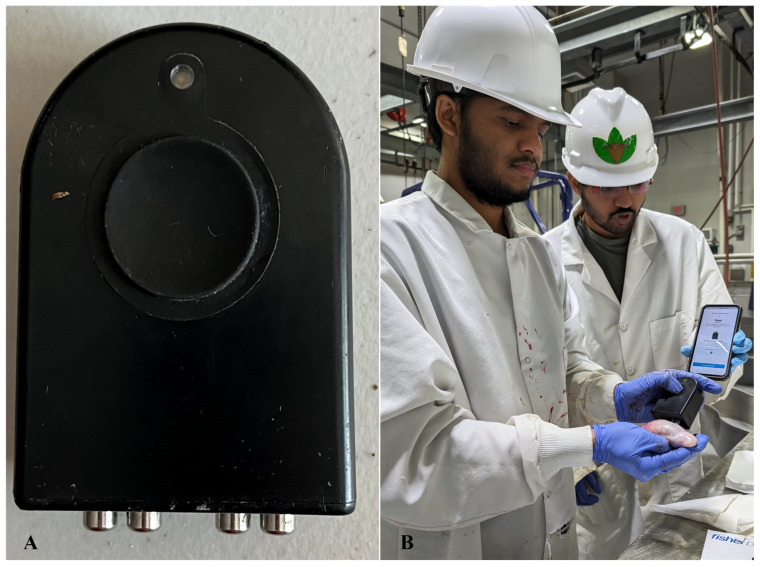
(**A**) Bioelectrical impedance analysis (BIA) device. (**B**) Application of BIA to goat testicles and recording BIA measurements using an online cloud-based server specifically designed by the BIA device provider.

At the end of the grazing period, the animals were slaughtered in the Fort Valley State University slaughter facility. Testicles and epididymides were collected from each animal and washed with warm sterile saline. BIA (Figure 2A) was applied to each testicle, and BIA measurements including Rs (Ω), parallel resistance (Rp, Ω), Xc, parallel reactance (Xcp, Ω), volume-specific reactance (Xcvol; Ω·cm^−3^), total impedance (Zp; Ω), and volume-specific impedance (Zpvol; Ω·cm^−3^) were recorded using an online cloud-based server specifically designed by the BIA device provider (Certified Quality foods, Clinton Twp, MI, USA, Figure 2B). The BIA unit consists of four electrodes: two signal electrodes and two detection electrodes (RJL Systems, Detroit, MI, USA). These electrodes are connected to an AC current of 800 µA and 50 kHz, and they produce voltage fluctuations ranging from 3.75 to 10.60 V. The electrodes used in data collection were made of stainless steel and were used to complete the circuit between the testis and the four electrodes. When the electrodes were in contact with the testis, the circuit was complete. For each testis, five consecutive measurements were taken. The mean values of these five technically replicated measurements were used for all subsequent statistical analyses to ensure consistency and reliability [20].

### 2.3. Measurement of Testicle and Epididymis Weights and Lengths

Collected testicles and epididymides from each buck were placed in separate plastic bags. Samples were then transported to the laboratory within 1 h of slaughter. Each testicle was then dissected, and the epididymis was separated using scalpel blades. The weights of the testicles and epididymides were measured in grams (g) by using a digital analytical balance (Mettler Toledo XS2002S; Mettler Toledo GmbH, Greifensee, Switzerland) with a maximum capacity of 2100 g and a sensitivity of 0.01 g. The testis length was measured in centimeters (cm) along the longitudinal axis of the testis, beginning from one pole of the testis to the other pole. Additionally, the length of the epididymides was measured in cm along their longitudinal axis, starting from the head and extending to the tail [21].

### 2.4. Collection and Processing of Epididymal Spermatozoa

For the collection of epididymal spermatozoa, each epididymis was washed three times with normal saline, followed by a final rinse with 70% ethyl alcohol, and then dried carefully with gauze. Multiple incisions were made in the tail of the epididymis using a sharp scalpel, and a gentle manual pressure was applied to release the spermatozoa in a Petri dish containing 3 mL of Andromed^®^ extender (Minitube, Verona, WI, USA) [22]. Sperm collected from both cauda epididymides of each animal were pooled and processed as a single sample. Samples were then evaluated for sperm motility, viability, morphology, and membrane and acrosome integrities.

### 2.5. Evaluation of Sperm Motility

Sperm motility was evaluated using computer-assisted sperm analysis (CASA; AndroScope^®^, Minitube, Verona, WI, USA) system. For analysis, a 5 µL aliquot of sperm suspension was loaded into a prewarmed (37 °C) Leja^®^ 20 µm deep counting chamber (Leja Products, Minitube, Verona, WI, USA). Slides were then examined under CASA, and the sperm motility was evaluated through AndroVision^®^ software (version 1.2.2, Minitube, Verona, WI, USA). A minimum of five random fields were captured per sample using a 20× phase-contrast objective lens. The CASA settings were optimized for buck spermatozoa and held constants for all analyses as follows: frame rate: 60 Hz; number of frames: 30; cell detection: minimum contrast of 35 and minimum cell size of 4 pixels. The static cell gate was set to 5.0 µm/s, and spermatozoa with a straightness (STR) ≥ 75% were classified as progressively motile [23].

### 2.6. Evaluation of Sperm Viability and Morphology

Sperm viability and morphology were evaluated by using eosin–nigrosin stain according to the method previously described [24]. A 20 µL sperm suspension was mixed with 10 µL eosin and 10 µL nigrosin stains on a pre-warmed glass slide. A thin smear was then prepared, air-dried, and examined under a phase contrast microscope at 40× magnification. Two hundred spermatozoa were counted, the percentage of live spermatozoa was determined, and the percentage of abnormal sperm was also recorded for each sample, as previously reported [24,25].

### 2.7. Membrane Integrity (Hypo-Osmotic Swelling; HOS) Test

The hypoosmotic swelling (HOS) test was used to assess the membrane integrity of spermatozoa according to the method described previously [26]. Briefly, 50 µL of the sperm suspension was mixed with 1 mL of pre-warmed 125 mOsm/L hypoosmotic solution. The hypoosmotic solution was prepared by dissolving 1.351 g of fructose and 0.753 g of sodium citrate dihydrate in 100 mL of sterile deionized water. The sperm and solution mixture was then incubated for 45 min at 37 °C. Fifteen microliters of the sperm–solution mixture was placed on a microscope slide, covered with a cover slip, and examined under a phase contrast microscope at 40× magnification. Two hundred spermatozoa were counted and examined for evidence of plasma membrane swelling, which indicates an intact plasma membrane (HOS+) [26]. The percentages of HOS+ spermatozoa were then recorded.

### 2.8. Evaluation of Sperm Acrosome Integrity

The Spermac (FertiPro N.V., Beernem, Belgium) stain was used to identify the spermatozoa’s acrosome integrity according to the method described by [20]. In short, air-dried sperm smear slides were fixed in a 10% formalin solution for 10 min. After that, each slide was stained at room temperature with Spermac solutions A, B, and C for 1 min each. The slides were kept dry and then examined under a phase contrast microscope (Fisher Scientific, Atlanta, GA, USA) at 100× magnification. Two hundred sperm cells were counted, and the percentages of sperm cells with a typical oval-shaped head, reddish pink post-acrosomal sections, and dark green anterior acrosome regions were calculated and recorded as sperm with intact acrosomes. Meanwhile, sperm with partially green heads and/or shed membranes were considered sperm with damaged acrosomes.

### 2.9. Statistical Analysis

Statistical analyses were performed in Python (version 3.10) using pandas, NumPy, and SciPy. All numeric variables were summarized using mean ± standard error of mean (SEM) and median with interquartile range (IQR). Healthy (H) and parasitized (P) groups were compared using the Mann–Whitney U test because several variables did not follow the assumptions of normality and homogeneity of variance. Effect sizes were quantified using Cliff’s delta (δ), a non-parametric measure of stochastic dominance ranging from −1 to +1. Precision of δ estimates was assessed using 2000 bootstrap resamples to generate bias-free 95% confidence intervals. Results for each variable, including descriptive statistics, U values, *p*-values, δ, and corresponding confidence intervals, were compiled and ranked by *p*-value. All significance tests were two-tailed, and analyses followed a fully non-parametric framework appropriate for heterogeneous physiological and impedance-based datasets. The correlation between BIA measurements, testicular morphometrics, and sperm quality parameters was determined by Pearson Correlation Coefficient using SAS (version 9.4); *p* < 0.05 was considered significant.

## 3. Results

### 3.1. Normality and Distributional Diagnostics

**Figure 3:**
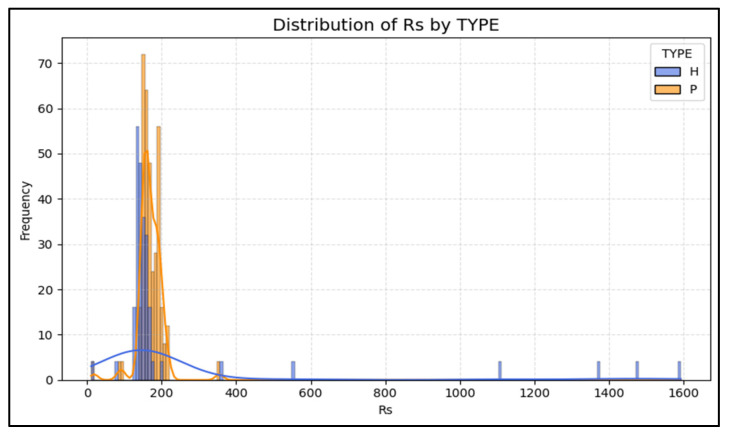
Distribution of serial resistance (Rs) in healthy (H) and parasitized (P) goats.

**Figure 4:**
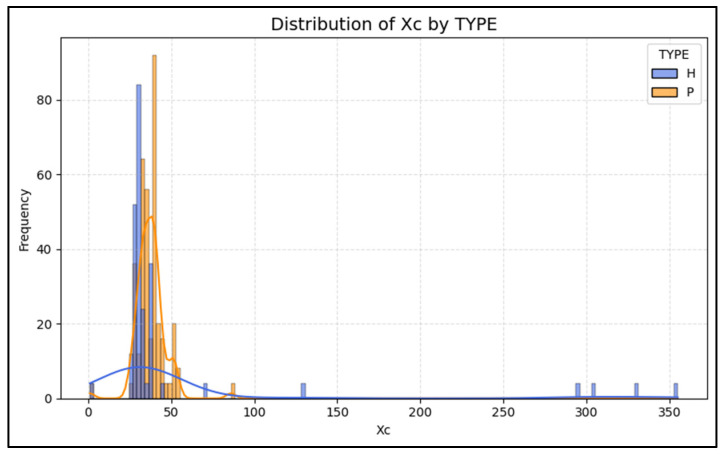
Distribution of reactance (Xc) in healthy (H) and parasitized (P) goats.

Every impedance trait produced highly significant *p*-values (*p* < 0.0001), rejecting the null hypothesis of equivalence. Cliff’s delta values quantify the magnitude of these differences without assuming normality. Values approaching ± 1.0 represent complete separation of distributions (Figure 3 and Figure 4), while near-zero values imply overlap. The non-parametric analysis of the collected data revealed that there was a near-total physiological divergence between groups, as well as a strong-to-moderate divergence for impedance traits (d ≈ 0.4–0.5).

### 3.2. Effect of Parasitic Infestation on Testicular Tissue Resistive Indices (Rp and Rs) as Detected by BIA

Both parallel (Rp) and series (Rs) resistance values were significantly lower (*p* < 0.0001) in parasitized goats compared to healthy goats. The effect size (d = −0.50 ± 0.09) demonstrates a medium-to-large downward shift in resistance across individuals (Table 1).

### 3.3. Effect of Parasitic Infestation on Testicular Tissue Reactance (Xc) and Phase-Dependent Indices

Reactance (Xc) decreased sharply in parasitized goats (*p* < 0.0001). Moreover, overall lower impedance (Zp) values were observed in parasitized goats as compared to the healthy goats (*p* < 0.0001), reflecting the combined decrease in resistive and capacitive components. However, volumetrically normalized indices (Zpvol and Xcvol) showed the opposite trend, being slightly higher in parasitized goats (d ≈ +0.44–0.45) (Table 1).

### 3.4. Effect of Parasitic Infestation on Goat Testicular and Epidydimal Weights and Lengths

Testicular and epididymal weights and lengths were significantly lower (*p* < 0.0001) in parasitized animals than in healthy animals, with a large effect size (Cliff’s d = 0.75–0.98). The mean epididymal weight was higher by over 25% in healthy goats, while testicular weight declined by ~29% in parasitized animals. A similar trend was noticed for testicular and epididymal lengths (Table 1).

### 3.5. Effect of Parasitic Infestation on Epididymal Sperm Quality Parameters

As shown in Table 2, sperm motility, viability, and membrane and acrosome integrities were significantly lower (*p* < 0.0001) in the parasitized goats than the healthy ones. On the other hand, total sperm abnormalities were significantly higher (*p* < 0.0001) in the parasitized group.

### 3.6. Correlations Between BIA Series Resistance (Rs) and Reactance (Xc), Testicular and Epididymal Morphometrics, and Sperm Quality Parameters

As shown in Table 3, a positive correlation was observed between testicular Rs and Xc and testis weight, testis length, epididymal length, sperm viability, and membrane integrity (*p* < 0.05). Moreover, testicular Xc had a positive correlation with sperm membrane integrity (*p* < 0.05). Significant positive correlations were also observed among testicular and epididymal weights and lengths, sperm motility, viability, and membrane and acrosome integrities (*p* < 0.05). Acrosome integrity was highly correlated with motility, viability, and membrane integrity.

## 4. Discussion

Parasitism imposes limitations and negatively impacts the output and welfare of affected animals, especially grazing species. In addition to depleting the resources of the afflicted animals, parasitism frequently causes a reduction in feed intake, which lowers productivity [27]. For that, the objective of our study was to employ BIA to assess the influence of parasitic infection on testicular morphometrics and sperm quality in goats.

Both Rp and Rs resistance values were significantly lower in parasitized goats. Biophysically, Rp and Rs represent the extracellular resistive pathways to current flow. Lower values thus indicate greater ionic conductance, consistent with tissue edema, plasma transudation, and cellular disorganization. It is well documented that parasitic infection promotes tissue inflammatory reaction and accumulation of interstitial fluid [28]. This occurs through the release of inflammatory cytokines and vascular permeability factors such as IL-6 and TNF-α [28]. These events can replace compact seminiferous architecture with an ion-rich extracellular milieu, thereby reducing resistivity. Additionally, necrotic and apoptotic germ cells release intracellular electrolytes, further lowering impedance. Hence, the observed drop in Rs and Rp directly reflects microscopic destruction of germinal epithelium and altered hydration dynamics in the testicular and epididymal tissues of parasitized bucks. Reactance (Xc), a measure of the capacitive storage of electrical energy across intact cell membranes, decreased sharply in parasitized goats. The reduction in Xc values in infected goats denotes loss of cellular integrity, reduced membrane capacitance, and diminished mitochondrial density [29]. Biologically, it signals atrophy of seminiferous tubules, disruption of tight junctions, and degeneration of spermatogenic cells. Similarly, in reproductive tissue, this translates into decreased sperm production and reduced viability. Based on previous studies, Xc depends on both membrane lipid composition and intracellular ionic gradients. Chronic parasitic infection likely alters phospholipid turnover and causes lipid peroxidation, reducing membrane dielectric strength [30]. This corresponds to well-documented oxidative bursts in nematode- or coccidia-challenged ruminants, where increased reactive oxygen species (ROS) cause irreversible peroxidative injury to the germinal epithelium [31]. Contrary to the current findings, we previously found that Rs and Xc values were higher in parasitized goats than in healthy ones [21]. This discrepancy could be ascribed to the differences in the tissue composition, as the latest study measured BIA values in the tail and ear parts of a live goat, which had different composition and water content profiles than the testes. This later study proves that the BIA technique can be safely applied to live animals to evaluate their health. Further studies investigating the association of BIA measures with semen quality and fertility in live goats are currently being carried out in our laboratory.

Overall lower impedance (Zp) values were observed in parasitized goats as compared to the healthy ones, reflecting the combined decrease in resistive and capacitive components. However, volumetrically normalized indices (Zpvol, Xcvol) showed the opposite trend, being slightly higher in parasitized goats (d ≈ +0.44–0.45). This paradox arises because, as gonadal volume declines due to atrophy, the impedance per unit volume appears artificially elevated. Physiologically, this represents the classic signatural pattern of degenerative remodeling, as it implies that structural miniaturization intensifies the relative dielectric heterogeneity of the remaining tissue [32].

In the current study, testicular and epididymal weights and lengths were higher in the healthy goats than in the parasitized ones. The pronounced reductions in testis and epididymal dimensions observed in parasitized goats represent a visible sign of chronic metabolic reallocation under sustained parasitic burden. Helminthic and protozoan infections impose persistent catabolic pressure by diverting host nutrients toward immune response and parasite maintenance [33]. This leads to protein-energy malnutrition, hypoproteinemia, and anemia, conditions that are well known to compromise the hypothalamic–pituitary–gonadal (HPG) axis [33]. The effects of parasitic infestation on the reproductive health of small ruminants have been previously reported [34,35]. For instance, previous studies reported a reduction in scrotal circumference in parasitized rams compared with healthy ones [35]. It is well known that scrotal circumference is associated with testicular morphometrics and the males’ reproductive performance [34]. Moreover, the negative impacts of parasitism on testicular morphometrics could be ascribed to the adverse influence of parasitic infection on animal body weight, general health, and performance [7]. Furthermore, chronic parasitism can exert deleterious effects on the host’s metabolic resources, leading to symptoms such as hypoproteinemia and hormonal dysregulation [36]. The synthesis of testosterone is critically dependent on the normal functioning of Leydig cells, regulated by luteinizing hormone (LH), which is an integral part of the HPG axis [37]. In contrast to our findings, a previous study reported no significant effects of parasitic infection on ram’s testicular weight and volume as compared with the control group [38]. These differences between our findings and the findings of Zully et al. (2020) [38] could be due to species differences, the parasitic load, different management systems, and the grazing area’s parasitic load.

It is well known that male fertility is highly associated with high sperm quality parameters. Herein, we found that sperm motility, viability, membrane integrity and acrosome intactness values were lower in parasitized goats than in healthy ones (Table 2). The deleterious effects of parasitic infestation on ejaculated sperm quality have been reported in various species, including camels and sheep [9,39,40,41,42]. The direct mechanisms by which parasites can impact gonadal health, sperm quality, and male fertility are still unknown; however, various notions have been explored, including the ability of parasites to produce biologically active substances like proteases and phospholipase A [43,44,45]. These substances can alter nutritional status and induce hyperthermia [43,44,45]. In rats, previous studies showed that infection with Trypanosoma brucei resulted in a defect in gonadotropic hormone secretion that is correlated with the proteases produced by the parasites [46].

In the present study, we found a strong correlation between Rs, Xc, testicular and epididymal morphometrics, sperm viability, and membrane and acrosome integrities in both parasitized and healthy goats (Table 3). Greater cellular integrity, tissue density, and hydration, all traits of fully formed testes and epididymides, are reflected in higher reactance values. This is consistent with earlier research that linked tissue health and BIA measurements [10,19]. Higher Xc may also be a sign of healthier sperm with intact membranes and acrosomes because it represents cell membrane capacitance—the ability of the insulating cell membranes to store electrical charge. This proves the association of BIA values and sperm quality in males.

## 5. Conclusions

In conclusion, parasitic infection was associated with low sperm quality parameters in male goats because it adversely affected testicular and epididymal morphometrics. Furthermore, testicular BIA values were significantly lower in parasitized animals, which is associated with a reduction in testis and epididymal weights and lengths, as well as compromised sperm motility, viability, and membrane and acrosome integrities. Future studies should focus on elucidating the physiological mechanisms linking parasitic infection to the observed changes in testicular bioelectrical properties.

## Figures and Tables

**Table 1:** Comparison of impedance traits and testicular morphometrics between healthy and parasitized goats.

Variables	Healthy Mean ± SEM	Healthy Median (IQR)	Parasitized Mean ± SEM	Parasitized Median (IQR)	*p*-Value	Cliff’s (δ)	(δ) 95% Low	(δ) 95% High
Rp (Ω)	247.26 ± 21.28	153.00 (21.00)	178.04 ± 1.82	173.00 (36.00)	<0.0001	−0.50	−0.58	−0.41
Rs (Ω)	235.21 ± 20.21	145.00 (23.00)	169.41 ± 1.76	165.00 (35.00)	<0.0001	−0.50	−0.58	−0.41
Zp (Ω)	50.61 ± 4.54	30.00 (6.00)	36.49 ± 0.45	36.00 (7.00)	<0.0001	−0.45	−0.53	−0.36
Zpvol (Ω·cm^−3^)	97.85 ± 9.69	87.00 (16.00)	113.67 ± 20.51	71.50 (15.00)	<0.0001	0.45	0.36	0.53
Xcvol (Ω·cm^−3^)	95.70 ± 9.53	85.00 (17.00)	111.96 ± 20.50	70.00 (15.00)	<0.0001	0.44	0.35	0.52
Xc (Ω)	52.08 ± 4.68	30.00 (8.00)	37.56 ± 0.48	37.00 (7.00)	<0.0001	−0.43	−0.52	−0.35
Xcp (Ω)	1159.44 ± 101.19	691.00 (215.00)	821.21 ± 7.84	797.50 (107.00)	<0.0001	−0.37	−0.47	−0.27
Testicular weight (gm)	134.74 ± 0.89	138.28 (22.72)	95.68 ± 0.95	89.23 (24.38)	<0.0001	0.90	0.87	0.92
Testicular length (cm)	15.47 ± 0.11	16.00 (2.20)	12.15 ± 0.07	11.75 (2.40)	<0.0001	0.88	0.84	0.92
Epididymal weight (gm)	23.98 ± 0.26	22.45 (0.43)	17.71 ± 0.10	17.51 (2.26)	<0.0001	0.98	0.96	0.99
Epididymal length (cm)	14.37 ± 0.07	13.90 (1.10)	12.80 ± 0.05	12.50 (1.35)	<0.0001	0.75	0.70	0.81

Rp (Ω): parallel resistance; Rs (Ω): series resistance; Zp (Ω): total impedance; Zpvol (Ω·cm^−3^): volume-specific impedance; Xcvol (Ω·cm^−3^): volume-specific reactance; Xc (Ω): reactance; Xcp (Ω): parallel reactance; IQR: interquartile range.

**Table 2:** Comparison of sperm quality parameters between healthy and parasitized goats.

Sperm Quality Parameters (%)	Healthy Mean ± SEM	Healthy Median (IQR)	Parasitized Mean ± SEM	Parasitized Median (IQR)	*p*-Value	Cliff’s (δ)	(δ) 95% Low	(δ) 95% High
Motility	59.88 ± 1.93	70.00 (52.50)	53.78 ± 1.31	60.00 (30.00)	<0.0001	0.17	0.07	0.26
Viability	63.58 ± 1.39	70.00 (37.00)	60.60 ± 1.15	65.00 (37.25)	<0.0001	0.09	−0.01	0.18
Total abnormalities	22.55 ± 0.34	22.00 (8.00)	27.76 ± 0.43	28.00 (13.00)	<0.0001	−0.39	−0.47	−0.31
Membrane integrity (HOS+)	64.50 ± 1.32	69.50 (29.00)	59.00 ± 1.21	62.00 (38.00)	<0.0001	0.15	0.06	0.24
Acrosome integrity	49.79 ± 0.89	48.50 (21.25)	40.08 ± 0.75	39.00 (22.00)	<0.0001	0.37	0.29	0.45

HOS: Hypo-osmotic swelling test. IQR: interquartile range.

**Table 3:** Correlation between BIA series resistance (Rs) and reactance (Xc), testicular and epididymal weights and lengths, sperm motility, viability, total abnormalities, and membrane and acrosome integrities.

	Resistance (Rs, Ω)	Reactance (Xc, Ω)	Testicular Weight	Testicular Length	Epididymal Weight	Epididymal Length	Motility	Viability	Total Abnormalities	Membrane Integrity (HOS+)	Acrosome Integrity
Resistance (Rs, Ω)	1.00	0.96	0.19	0.16	0.07	0.16	0.05	0.20	−0.06	0.15	0.14
*p*-value		<0.0001	0.01	0.03	0.38	0.04	0.46	0.01	0.40	0.05	0.07
Reactance (Xc, Ω)	0.96	1.00	0.18	0.16	0.07	0.16	0.07	0.21	−0.07	0.18	0.16
*p*-value	<0.0001		0.02	0.03	0.36	0.03	0.39	0.007	0.37	0.02	0.03
Testicular weight	0.19	0.18	1.00	0.76	0.79	0.56	0.58	0.13	−0.02	0.29	0.32
*p*-value	0.01	0.02		<0.0001	<0.0001	<0.0001	<0.0001	0.11	0.76	0.0002	<0.0001
Testicular length	0.16	0.16	0.76	1.00	0.71	0.70	0.54	0.23	−0.15	0.43	0.37
*p*-value	0.03	0.03	<0.0001		<0.0001	<0.0001	<0.0001	0.003	0.05	<0.0001	<0.0001
Epididymal weight	0.05	0.05	0.56	0.65	1.00	0.82	0.48	0.13	0.04	0.34	0.38
*p*-value	0.48	0.50	<0.0001	<0.0001		<0.0001	<0.0001	0.09	0.61	<0.0001	<0.0001
Epididymal length	0.16	0.16	0.66	0.65	0.71	1.00	0.33	0.06	−0.10	0.14	0.21
*p*-value	0.04	0.03	<0.0001	<0.0001	<0.0001		<0.0001	0.46	0.20	0.07	0.008
Motility	0.05	0.07	0.58	0.42	0.56	0.48	1.00	0.59	0.007	0.48	0.80
*p*-value	0.46	0.39	<0.0001	<0.0001	<0.0001	<0.0001		<0.0001	0.9264	<0.0001	<0.0001
Viability	0.20	0.21	0.13	0.06	0.15	0.13	0.59	1.00	−0.01	0.58	0.61
*p*-value	0.01	0.007	0.11	0.46	0.06	0.09	<0.0001		0.80	<0.0001	<0.0001
Total abnormalities	−0.06	−0.07	−0.02	−0.20	−0.001	−0.04	−0.007	−0.01	1.00	0.13	0.03
*p*-value	0.40	0.37	0.76	0.009	0.98	0.61	0.92	0.80		0.09	0.64
Membrane integrity (HOS+)	0.15	0.18	0.29	0.09	0.31	0.34	0.48	0.58	0.13	1.00	0.53
*p*-value	0.05	0.02	0.0002	0.23	<0.0001	<0.0001	<0.0001	<0.0001	0.09		<0.0001
Acrosome integrity	0.14	0.16	0.32	0.21	0.44	0.38	0.80	0.61	0.03	0.53	1.00
*p*-value	0.07	0.03	<0.0001	0.008	<0.0001	<0.0001	<0.0001	<0.0001	0.64	<0.0001	

*p* < 0.05 was considered significant. HOS: hypo-osmotic swelling test.

## Data Availability

All generated data are provided in this submission.

## Abbreviations

The following abbreviations are used in this manuscript:

BIA	Bioelectrical Impedance
Rp	Parallel Resistance
Rs	Series Resistance
Zp	Total Impedance
Zpvol	Volume-Specific Impedance
Xcvol	Volume-Specific Reactance
Xc	Reactance
Xcp	Parallel Reactance
HOS	Hypo-osmotic Swelling

## References

[1] Mazhangara I.R., Chivandi E., Mupangwa J.F., Muchenje V. (2019). The Potential of Goat Meat in the Red Meat Industry. Sustainability.

[2] Nyaupane N., Gillespie J., Mcmillin K. (2016). The Marketing of Meat Goats in the US: What, Where, and When?. J. Food Distrib. Res..

[3] Coop I.E. (1982). Sheep and Goat Production.

[4] Delgadillo J.A., Malpaux B., Chemineau P. (1997). La Reproduction Des Caprins Dans Les Zones Tropicales et Subtropicales. INRA Prod. Anim..

[5] Ghimire T.R., Bhattarai N. (2019). A Survey of Gastrointestinal Parasites of Goats in a Goat Market in Kathmandu, Nepal. J. Parasit. Dis..

[6] Papadopoulos E., Gallidis E., Ptochos S. (2012). Anthelmintic Resistance in Sheep in Europe: A Selected Review. Vet. Parasitol..

[7] Schulte-Hostedde A.I., Millar J.S. (2004). Intraspecific Variation of Testis Size and Sperm Length in the Yellow-Pine Chipmunk (*Tamias amoenus*): Implications for Sperm Competition and Reproductive Success. Behav. Ecol. Sociobiol..

[8] Møller A.P. (1989). Ejaculate Quality, Testes Size and Sperm Production in Mammals. Funct. Ecol..

[9] Rouatbi M., Gharbi M., Rjeibi M.R., Salem I.B., Akkari H., Lassoued N., Rekik M. (2016). Effect of the Infection with the Nematode *Haemonchus contortus* (Strongylida: Trichostrongylidae) on the Haematological, Biochemical, Clinical and Reproductive Traits in Rams. Onderstepoort J. Vet. Res..

[10] Tinsley G.M., Harty P.S., Moore M.L., Grgic J., Silva A.M., Sardinha L.B. (2019). Changes in Total and Segmental Bioelectrical Resistance Are Correlated with Whole-Body and Segmental Changes in Lean Soft Tissue Following a Resistance Training Intervention. J. Int. Soc. Sports Nutr..

[11] Gauly M., Schackert M., Hoffmann B., Erhardt G. (2006). Influence of Sex on the Resistance of Sheep Lambs to an Experimental *Haemonchus contortus* Infection. Dtsch. Tierarztl. Wochenschr..

[12] Sánchez-Iglesias A., Fernández-Lucas M., Teruel J.L. (2012). The Electrical Basis of Bioimpedance. Nefrología.

[13] Norman K., Stobäus N., Pirlich M., Bosy-Westphal A. (2012). Bioelectrical Phase Angle and Impedance Vector Analysis—Clinical Relevance and Applicability of Impedance Parameters. Clin. Nutr..

[14] Mulasi U., Kuchnia A.J., Cole A.J., Earthman C.P. (2015). Bioimpedance at the Bedside: Current Applications, Limitations, and Opportunities. Nutr. Clin. Pract..

[15] Piccoli A., Pastori G., Codognotto M., Paoli A. (2007). Equivalence of Information from Single Frequency v. Bioimpedance Spectroscopy in Bodybuilders. Br. J. Nutr..

[16] Gonzalez M.C., Barbosa-Silva T.G., Bielemann R.M., Gallagher D., Heymsfield S.B. (2016). Phase Angle and Its Determinants in Healthy Subjects: Influence of Body Composition. Am. J. Clin. Nutr..

[17] Liprino A., Giacone F., Lombardo D., Asmundo M.G., Russo G.I., Abdelhameed A.S., Cimino S., Guglielmino A., Chamayou S. (2024). Phase Angle at Bioelectric Impedance Analysis Is Associated with Detrimental Sperm Quality in Idiopathic Male Infertility: A Preliminary Clinical Study. Front. Endocrinol..

[18] Soliman S.M., Salem H.M., Ahmed A.E., Saad A., El-Saadony M.T. (2024). *Haemonchus contortus* Infection of Small Ruminants and the Use of Garlic as an Anthelmintic Natural Alternative: An Updated Review. J. Hell. Vet. Med. Soc..

[19] Siddique A., Batchu P., Shaik A., Gurrapu P., Erukulla T.T., Ellington C., Rubio Villa A.L., Brown D., Mahapatra A., Panda S. (2025). Evaluating the Efficacy of Bioelectrical Impedance Analysis Using Machine Learning Models for the Classification of Goats Exposed to Haemonchosis. Front. Vet. Sci..

[20] Siddique A., Herron C.B., Valenta J., Garner L.J., Gupta A., Sawyer J.T., Morey A. (2022). Classification and Feature Extraction Using Supervised and Unsupervised Machine Learning Approach for Broiler Woody Breast Myopathy Detection. Foods.

[21] Oeyemi M.O., Fayomi A.P., Adejoke A.D., Mary O.K. (2012). Testicular and Epididymal Parameters of Sahel Buck in the Humid Zone of Nigeria. Int. J. Morphol..

[22] Abu A.H., Kisani A.I., Ahemen T. (2016). Evaluation of Sperm Recovered after Slaughter from Cauda Epididymides of Red Sokoto Bucks. Vet. World.

[23] Singh A.K., Kumar A., Bisla A. (2021). Computer-Assisted Sperm Analysis (CASA) in Veterinary Science: A Review. Indian J. Anim. Sci..

[24] Ghallab A., Fadl A.M., Moawad A.R., Abdel D., El-Badry M. (2019). Optimization of the Protocol for Cryopreservation of Arabian Stallion Spermatozoa: Effects of Centrifugation, Semen Extenders and Cryoprotectants. Cryoletters.

[25] Ghallab A.R.M., Shahat A.M., Fadl A.M., Ayoub M.M., Moawad A.R. (2017). Impact of Supplementation of Semen Extender with Antioxidants on the Quality of Chilled or Cryopreserved Arabian Stallion Spermatozoa. Cryobiology.

[26] Fonseca J.F., Torres C.A.A., Maffili V.V., Borges A.M., Santos A.D.F., Rodrigues M.T., Oliveira R.F.M. (2005). The Hypoosmotic Swelling Test in Fresh Goat Spermatozoa. Anim. Reprod..

[27] Fthenakis G.C., Papadopoulos E. (2018). Impact of Parasitism in Goat Production. Small Rumin. Res..

[28] M’bondoukwé N.P., Moutongo R., Gbédandé K., Ngomo J.M.N., Hountohotegbé T., Adamou R., Lengongo J.V.K., Bello K.P., Mawili-Mboumba D.P., Luty A.J.F. (2022). Circulating IL-6, IL-10, and TNF-Alpha and IL-10/IL-6 and IL-10/TNF-Alpha Ratio Profiles of Polyparasitized Individuals in Rural and Urban Areas of Gabon. PLoS Negl. Trop. Dis..

[29] Goswami I., Perry J.B., Allen M.E., Brown D.A., von Spakovsky M.R., Verbridge S.S. (2018). Influence of Pulsed Electric Fields and Mitochondria-Cytoskeleton Interactions on Cell Respiration. Biophys. J..

[30] Ray S., Kassan A., Busija A.R., Rangamani P., Patel H.H. (2016). The Plasma Membrane as a Capacitor for Energy and Metabolism. Am. J. Physiol. Cell Physiol..

[31] Pawłowska M., Mila-Kierzenkowska C., Szczegielniak J., Woźniak A. (2024). Oxidative Stress in Parasitic Diseases—Reactive Oxygen Species as Mediators of Interactions between the Host and the Parasites. Antioxidants.

[32] Well D., Yang H., Houseni M., Iruvuri S., Alzeair S., Sansovini M., Wintering N., Alavi A., Torigian D.A. (2007). Age-Related Structural and Metabolic Changes in the Pelvic Reproductive End Organs. Semin. Nucl. Med..

[33] Fekete S.G., Kellems R. (2007). Interrelationship of Feeding with Immunity and Parasitic Infection: A Review. Vet. Med..

[34] Gouletsou P.G., Fthenakis G.C. (2010). Clinical Evaluation of Reproductive Ability of Rams. Small Rumin. Res..

[35] Ridler A.L., Smith S.L., West D.M. (2012). Ram and Buck Management. Anim. Reprod. Sci..

[36] Singla L.D., Sumbria D., Sudan V., Kaur P. (2024). Impact of Parasitic Infections on Host Metabolism: An Overview. Indian J. Anim. Res..

[37] Walker W.H., Cheng J. (2005). FSH and Testosterone Signaling in Sertoli Cells. Reproduction.

[38] Zully H.-R., Nelson V., Daniel F.-A. (2020). Effect of Gastrointestinal Nematodes in Ram Sperm Production. GSC Adv. Res. Rev..

[39] Al-Qarawi A.A., Omar H.M., Abdel-Rahman H.A., El-Mougy S.A., El-Belely M.S. (2004). Trypanosomiasis-Induced Infertility in Dromedary (*Camelus dromedarius*) Bulls: Changes in Plasma Steroids Concentration and Semen Characteristics. Anim. Reprod. Sci..

[40] Folstad I., Skarstein P. (1997). Is Male Germ Line Control Creating Avenues for Female Choice?. Behav. Ecol..

[41] Måsvær M., Liljedal S., Folstad I. (2004). Are Secondary Sex Traits, Parasites and Immunity Related to Variation in Primary Sex Traits in the Arctic Charr?. Proc. R. Soc. B Biol. Sci..

[42] Sekoni V.O. (1992). Effect of Trypanosoma Vivax Infection on Semen Characteristics of Yankasa Rams. Br. Vet. J..

[43] Rhodes A.P. (1975). Seminal Degeneration Associated with Chorioptic Mange of the Scrotum of Rams. Aust. Vet. J..

[44] Sekoni V.O., Rekwot P.I., Bawa E.K. (2004). Effects of *Trypanosoma vivax* and *Trypanosoma congolense* Infections on the Reaction Time and Semen Characteristics of Zebu (Bunaji) × Friesian Crossbred Bulls. Theriogenology.

[45] Tizard I., Nielsen K.H., Seed J.R., Hall J.E. (1978). Biologically Active Products from African Trypanosomes. Microbiol. Rev..

[46] Hublart M., Tetaert D., Croix D., Boutignon F., Degand P., Boersma A. (1990). Gonadotropic Dysfunction Produced by *Trypanosoma brucei brucei* in the Rat. Acta Trop..

